# Exploring the Role of Gut Bacteria in Health and Disease in Preterm Neonates

**DOI:** 10.3390/ijerph17196963

**Published:** 2020-09-23

**Authors:** Jimmy Kok-Foo Lee, Loh Teng Hern Tan, Amutha Ramadas, Nurul-Syakima Ab Mutalib, Learn-Han Lee

**Affiliations:** 1 Novel Bacteria and Drug Discovery Research Group (NBDD), Microbiome and Bioresource Research Strength (MBRS), Jeffrey Cheah School of Medicine and Health Sciences, Monash University Malaysia, Bandar Sunway 47500, Selangor Darul Ehsan, Malaysia; jimmy.lee@monash.edu (J.K.-F.L.); loh.teng.hern@monash.edu (L.T.H.T.); amutha.ramadas@monash.edu (A.R.); 2 Clinical School Johor Bahru, Jeffrey Cheah School of Medicine and Health Sciences, Monash University Malaysia, Johor Bahru 80100, Malaysia; 3 UKM Medical Molecular Biology Institute (UMBI), UKM Medical Centre, Universiti Kebangsaan Malaysia, Kuala Lumpur 56000, Malaysia

**Keywords:** gut bacteria, gut microbiome, preterm neonates, mortality, dysbacteriosis

## Abstract

The mortality rate of very preterm infants with birth weight <1500 g is as high as 15%. The survivors till discharge have a high incidence of significant morbidity, which includes necrotising enterocolitis (NEC), early-onset neonatal sepsis (EONS) and late-onset neonatal sepsis (LONS). More than 25% of preterm births are associated with microbial invasion of amniotic cavity. The preterm gut microbiome subsequently undergoes an early disruption before achieving bacterial maturation. It is postulated that bacterial gut colonisation at birth and postnatal intestinal dysbacteriosis precede the development of NEC and LONS in very preterm infants. In fact, bacterial colonization patterns in preterm infants greatly differ from term infants due to maternal chorioamnionitis, gestational age, delivery method, feeding type, antibiotic exposure and the environment factor in neonatal intensive care unit (NICU). In this regard, this review provides an overview on the gut bacteria in preterm neonates’ meconium and stool. More than 50% of preterm meconium contains bacteria and the proportion increases with lower gestational age. Researchers revealed that the gut bacterial diversity is reduced in preterm infants at risk for LONS and NEC. Nevertheless, the association between gut dysbacteriosis and NEC is inconclusive with regards to relative bacteria abundance and between-sample beta diversity indices. With most studies show a disruption of the Proteobacteria and Firmicutes preceding the NEC. Hence, this review sheds light on whether gut bacteria at birth either alone or in combination with postnatal gut dysbacteriosis are associated with mortality and the morbidity of LONS and NEC in very preterm infants.

## 1. Introduction

Prematurity is the leading cause of global under-5 deaths from the year 2015 [1]. Preterm babies are born from 22 weeks to 36 weeks 6 days of gestational age. They account for 11.1% of 135 million live births in the world in 2010 [2]. The care of preterm infants is both labour intensive and consumes a major portion of neonatal intensive care unit (NICU) resources [3]. The very preterm infants born less than 32 weeks gestation comprises 15.6% of preterm births and have a high risk of mortality and morbidity [4]. It was reported that the survival till discharge of less than 80% for the very preterm infants and very low birth weight (VLBW) infants in Malaysia [5]. The survival of very preterm infants under the birth weight category of 500 to <1000 g and 1000 to <1500 g were 53.4% and 89.7%, respectively. Meanwhile, the VLBW infants in Canada achieve survival rates of 85.5% and 97.5% for the two birth weight categories [6].

The survivors of infants born VLBW (<1500 g) and especially the extremely low birth weight (ELBW) (<1000 g) are at a high risk of serious pre-discharge morbidities. These morbidities include early and late-onset neonatal sepsis (LONS), necrotising enterocolitis (NEC), poor somatic growth besides intraventricular haemorrhage, periventricular leukomalacia, retinopathy of prematurity, patent ductus arteriosus and chronic lung disease. The Canadian Neonatal Network [6] reported 67% survivors without any of the 6 morbidities of severe neurological injury [(Intraventricular haemorrhage (IVH) III & IV, periventricular leukomalacia (PVL)], retinopathy of prematurity (ROP) 3 & 4, oxygen use at 36 weeks or discharge, patent ductus arteriosus (PDA) requiring surgical ligation, NEC stage 2 or 3 and culture proven early-onset neonatal sepsis (EONS) and LONS. There is no reliable Malaysian data on the serious pre-discharge morbidities in ELBW and VLBW infants.

There is evidence of microbial intra-uterine invasion in 25 to 40% of preterm births [7,8] and 7 to 12% of preterm labour with intact membranes [9]. The polymerase chain reaction (PCR) technique using 16S rDNA for identification of bacteria can demonstrate the prevalence of microbial invasion of amniotic fluid to be 30 to 50% higher than conventional culture-based method [10,11]. The amniotic fluid of preterm births can be colonised by the ascending vaginal microbes even if the amniotic membrane is intact [11] and from placenta microbiome linked to the hypothesised haematogenous spread from maternal oral microbiome [12,13].

Maternal chorioamnionitis (bacterial infection of the amniotic fluid) together with fetal systemic inflammatory response syndrome [14] has been linked with numerous morbidities in very preterm neonates. These morbidities include an increase of PVL/Cerebral palsy [15,16], IVH [17,18,19], ROP [20], NEC [18,21] and EONS [19,22,23]. Chorioamnionitis is protective of LONS [23] probably through hastening the immune maturation. Chorioamnionitis is not associated directly with mortality in preterm infants [19,24] as the majority of the microbial invasion of the amniotic cavity are caused by low-virulence bacteria [11].

**Figure 1:**
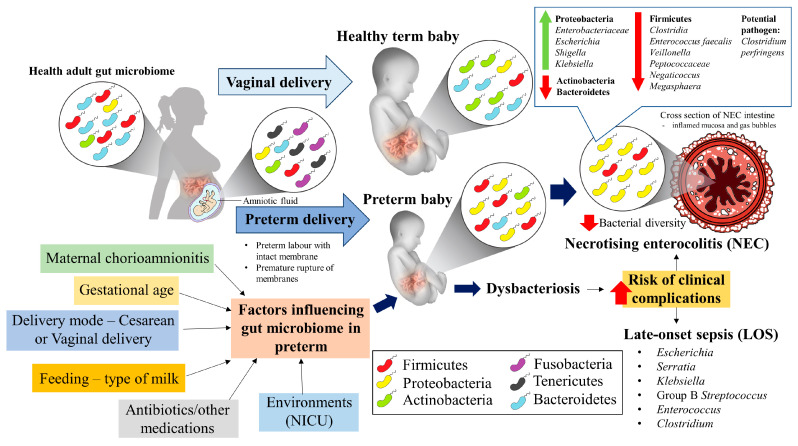
A graphical abstract illustrates the associations between the gut microbiome of preterm infants alone or in combination with postnatal gut dysbacteriosis and the predisposition to NEC and LONS.

After birth, an early balanced gut microbiome is crucial to physiological functions and immune system maturation [25]. In recent year, burgeoning of evidence has also pointed out the significance of gut microbiome in providing colonization resistance against pathogens or opportunistic gut-derived pathobionts. The disruption of the maturation of bacterial gut microbiome, namely intestinal dysbacteriosis predisposes the neonates to NEC [26] and nosocomial infection or LONS [27,28,29,30]. The poor somatic growth of very preterm infants is related to a disrupted gut-brain axis [31], distorted gut microbiota composition [32,33], inadequate calorie intake [34] and existing neonatal morbidities. Although numerous studies support the notion that intestinal bacteria play an essential role in the pathogenesis of NEC, EONS and LONS, there has been no single bacterial species consistently identified as the causative agent. That said, several microorganisms have been implicated in NEC, primarily from the phyla Firmicutes (coagulase-negative staphylococci) and Proteobacteria (*Cronobacter sakazakii*, *Klebsiella* sp. and *E. coli*) [35,36]. Specifically, an increased abundance of *Clostridium* spp. and associated toxins has also been observed in the stool of neonates with NEC [37,38,39]. Acquisition of Group B *Streptococcus* (GBS) and *E. coli* during the birth process has been identified as the primary cause of EONS within 3 days postpartum among preterm infants [40]. Meanwhile, commensals of the skin or intestines, including *Staphylococcus* spp., *E. coli*, *K. pneumoniae* or *Candida* spp. are typically causing the LONS [41]. Thus, the pattern and magnitude of the bacterial gut disruption is an active ongoing research subject in preterm infants with adverse outcomes. This review aims to provide valuable insights into the gut bacteria in preterm neonates’ meconium and stool, gut bacteria in NEC and LONS (Figure 1).

## 2. Gut Bacteria in Preterm Neonates—An Overview

In the healthy adult gut, the dominant phyla of Bacteroidetes and Firmicutes make up >90% of the microbiota [42]. The lesser phyla are Proteobacteria, Actinobacteria, Fusobacteria and Verrucomicrobia [43]. More than 99% of healthy gut microbes are anaerobes residing mainly in the distal ileum and colon. In comparison, the 4 main bacterial phyla present in the gut for both healthy neonate and preterm VLBW infant include Firmicutes, Proteobacteria, Actinobacteria and Bacteroidetes [44,45,46,47,48]. However, preterm and VLBW infants have lower microbial diversity, disrupted gut microbiome and increasing colonization of hospital acquired potentially pathogenic microorganisms when compared to age-matched full-term infants [49]. For instance, the GBS and *Escherichia coli* were the common cause of severe EOS and deaths in NICU [50,51].

Following birth, there are numerous factors which disrupt the initial gut bacteria colonisation and subsequent maturation of the preterm gut microbiome. Primarily, these factors include gestational age, mode of delivery (vaginal birth vs. Caesarean section), feeding methods (breast milk vs. formula) and early use of antibiotics [52,53]. For instance, Arboleya, et al. [47] demonstrated that preterm compared to term neonates have increased *Enterococcus*, *Enterobacter*, *Lactobacillus*, *Staphylococcus* and decreased *Bacteroides*, *Bifidobacterium* and *Atopobium*. Gut colonisation by *Bifidobacterium* has also shown to be delayed in preterm infants. A prospective study revealed that the gestational age has significant impact on gut colonization by bifidobacteria, whereby a birth at a gestational age < 33 weeks seems to impair bifidobacterial colonization and predisposes preterm infants to gut infection and diseases [54].

### 2.1. The Amniotic Fluid Is Colonised by Bacteria before Preterm Delivery

The conventional view that human fetus is sterile has been challenged by the recent evidence showing the presence of microorganisms in normal healthy placenta, amniotic fluid and meconium [55,56,57]. Despite that, there are studies questioning on the existence of a normal placental microbiome, thereby suggesting that the detection of bacteria in the placental samples may be due to contamination acquired during delivery, sample processing and analysis [58,59,60]. Nevertheless, prenatal seeding of the human microbiome is expected to have significant implications on the pregnancy outcomes and subsequently the post-natal colonization events [55,61]. It is hypothesized that the in utero colonization could trigger immune activation or sensitization, resulting in production of immune mediators, thus affecting the immune system, gut and brain of the fetus [55,61].

In contrary, the presence of pathogenic microorganisms in the intra-amniotic spaces has been well-known as the main driver for pro-inflammatory condition that can lead to adverse pregnancy outcomes like preterm birth and spontaneous death of fetus [62]. One of the major findings from a retrospective study revealed that a significant inverse association between the bacterial abundance in amniotic fluid and gestational age at delivery, thus supporting the contributory role for intra-uterine infection in preterm birth [10,63]. A systemic host inflammatory response induced possibly via oral periodontal disease and direct oral-utero transmission were the possible mechanisms suggested to enhance the adverse pregnancy outcomes [62].

Preterm parturition can occur with distinct clinical phenotypes, such as the preterm labor with intact membranes and preterm premature rupture of membranes (PROM). It has been long that microbial invasion of the amniotic cavity (MIAC) is associated with preterm PROM cases. PCR-based molecular studies identified that the predominant phyla in human gut microbiome Firmicutes, Bacteroidetes, Actinobacteria and Proteobacteria are present in the amniotic fluid during preterm labour [10,11,64]. Although at different relative proportions, the Fusobacteria and Tenericutes together made up slightly more than 50% of the bacteria in the amniotic fluid in both clinical scenario of preterm labour with intact membranes and preterm PROM [11]. Several specific genera from the phyla of Tenericutes (*Ureaplasma* and *Mycoplasma*) and Fusobacteria (*Sneathia* and *Leptotrichia*) were also suggested to appear as intra-amniotic pathogens due to their increased prevalence in cases of peripartum bacteremia [65,66]. Interestingly, a recent in vivo study established a causal link between *Ureaplasma* species and adverse pregnancy and neonatal outcomes, demonstrating that the intra-amniotic inoculation of clinically isolated *Ureaplasma* species induced preterm birth and neonatal mortality by causing severe inflammatory response in several reproductive organs of pregnant mice [67]. Taken together, these findings have indicated that the amniotic fluid from pregnant women who deliver prematurely, regardless of with intact or rupture membrane, is colonized with microorganisms.

### 2.2. The Bacteria Present in the Meconium

Generally, first-pass meconium samples are collected as a proxy for the bacterial communities in the in utero fetal gut environment. It is believed that meconium is a unique substance, consisting of bile acids, pancreatic secretions, epithelial cells, forming through the physiological process of in utero swallowing of amniotic fluid before birth [68]. Furthermore, meconium sample collection is simpler, more rapid and noninvasive when compared to the amniocentesis procedure of obtaining the amniotic fluid for the purpose of research investigations. That said, the analysis of meconium microbiome from infants can be essential to gain a better understanding of microbial establishment in human intestine as well as their potential role in infant health and disease.

Among preterm neonates, the gut bacteria present in meconium varies with gestation age. A study of 52 infants with gestation 23 to 41 weeks [69] revealed bacterial PCR was positive in 74% (of 35 infants) of neonates less than 33 weeks and 53% (of 17 infants) more than 33 weeks gestation. The mean relative abundance and standard deviation for gestation less than 33 weeks was Firmicutes 44.5 ± 17.6%, Proteobacteria 35.4 ± 17.9%, Actinobacteria 7.6 ± 7.6% and Bacteroidetes 6.0 ± 8.0%.

A similar pattern of bacteria phyla present in the meconium was demonstrated in 14 preterm neonates with gestation 24–31 weeks [70]. The most abundant phyla were Firmicutes 63.4% with 95%CI 42.2–84.6, Proteobacteria 27.7% with 95%CI 7.61–47.7 and Actinobacteria 3.5% with 95% CI 2.2–22.2. The main genera present in the meconium were *Bacilli*, *Staphylococcus*, *Streptococcus*, *Enterococcus* and *Lactobacillus* (all Firmicutes). The Shannon-Weaver diversity index of the meconium was 3.8 which increased to 4.0 for the stools at 3 weeks.

In a study on 23 neonates [71] showed that the Simpson diversity index of meconium in <30 weeks was a mean of 6.20 (sd 2.62) in 5 neonates compared to 8.56 (2.37) in 7 neonates for >7 days postnatal age. A longitudinal cohort study on 12 preterm with gestation 28.2 ± 1.5 weeks, birth weight 1055 ± 234 g showed that the Shannon diversity 1.4 is lowest at birth, and Tenericutes was present in the meconium [72].

Collectively, there has been overarching agreement in the literature that the bacterial composition of meconium reflects the in utero microbial environment. Studies showed that large relative abundance of genera from meconium was shared with the amniotic fluid samples than from other microbial niches, indicating that the microbes from the meconium of the infants are not of postnatal origin [69,71]. In general, the meconium of preterm baby is predominated by Firmicutes and Proteobacteria as well as low species diversity when compared to term baby [70].

### 2.3. The Maturation of the Gut Microbiome

There are two conceptual models to the development of the preterm gut microbiome. The first concept is the gut microbiome develops in a structured pattern which is dependent on the host maturation with little influence from the environmental factors [73,74]. The second concept is the external factors such as diet [75], antibiotic exposure [76,77] and hospital environment [72,78] play a major role in the gut microbiome assembly.

A study by La Rosa, et al. [73] investigated on 58 preterm with median gestation 27.1 weeks and median birthweight 960 (IQR 800, 1200) inclusive of 7 neonates with septicaemia, showed there were abrupt shifts of the gut bacteria classes but with nonrandom assembly pattern. The saltatory change progresses from the slow decrease of Bacilli (Firmicutes), while Gammaproteobacteria (Proteobacteria) bloomed and remained abundant, and followed by gradual increase of Clostridia (Firmicutes) in the gut microbiome. The proportion of Bacilli-Gammaproteobacteria-Clostridia in infancy was 19%–54%–18.5%. Clostridia was well represented by 33–36 weeks corrected age. Overall, the study suggested that the microbial population changes were mainly influenced by gestation at birth of the neonates.

Grier, et al. [74] showed a similar pattern with 95 preterm babies with gestation 28.8 ± 3.4 weeks and 25 term infants. The gut microbiota of subjects across postmenstrual age from 24 to 46 weeks were analysed. The relative abundance of the classes was Bacilli (Firmicutes) 41.8%, Gammaproteobacteria (Proteobacteria) 23.0%, Clostridia (Firmicutes) 22.5%, Actinobacteria (Actinobacteria) 6.5% and Bacteroidia (Bacteroidetes) 5.1%. There was a patterned progression with predominance of Bacilli (Firmicutes), Gammaproteobacteria (Proteobacteria) and Clostridia (Firmicutes) from gestational age 27 weeks to 30 weeks.

A study on 32 VLBW infants which included 3 NEC and 8 LONS and 21 healthy controls [76] supported the notion that the microbiome development was individualised and not influenced by NEC and LONS. Most stools were dominated by *Enterobacteriaceae*, *Enterococcus*, *Staphylococcus* and *Bacteroides* with reduction of *Bifidobacterium* and this reflected antibiotic use. One to 3 genera of bacteria dominated most stool samples. Each individual patient accounted for 48% (*p* < 0.001) of the variance in the stool samples from permutational multivariate analysis of variance (PERMANOVA) while the delivery mode accounted for 12% of the variance (*p* < 0.05).

### 2.4. The Predominant Bacteria in the Early Stools

The early colonisers are facultative anaerobes which will be replaced by the obligate anaerobes Bacteroides and Clostridia by 1 to 2 weeks of life. Chernikova, et al. [77] did a longitudinal study on weekly stools of 9 extremely preterm neonates with gestation 24–29 weeks. The facultative anaerobes *Escherichia* (*p* = 0.03) and *Shigella* (*p* = 0.027) decreased over time. *Streptococcus* (*p* < 0.001) and *Enterococcus* (*p* = 0.004) increased over time. *Staphylococcus* had the most significant decrease over time (*p* = 0.006) and *Veillonella* (*p* < 0.001) the most significant increase over time. The dominant genera were *Enterobacter* 19%, *Bacteroides* 14% and *Staphylococcus* 12%. There was a paucity of *Bifidobacterium* 0.01% and *Escherichia* 0.17% in this cohort.

The cohort by Ho, et al. [79] on 45 VLBW infants with gestation 27.9 ± 2.2 weeks and birthweight 1126 ± 208 g showed a dichotomy of gut microbiome. A group started with low Gammaproteobacteria which increased with time with concomitant decreasing Firmicutes. The other group had high Gammaproteobacteria and low Firmicutes throughout. Meanwhile, Actinobacteria and Bacteroidetes were low throughout for both groups.

### 2.5. Proportion of Bacteria and Bacterial Diversity in the First Month of Life in Preterm Infants

Drell, et al. [80] studied 50 preterm with gestation 24–31 weeks, 2 died, 2 EONS, 2 LONS, 7 NEC stage ≥2. The stool was collected at 1 week, 1 month and 2 months. The ELBW gut was dominated by facultative anaerobes and potential pathogens such as *Staphylococcus* and *Enterobacteriaceae*, and lacked the beneficial *Bacteroides*, *Bifidobacterium* and *Lactobacillus*. Proteobacteria had a prevalence of 94% and mean relative abundance 0.51 (sd = 0.42). Its prevalence increased from 0.28 (sd = 0.44) at 1 week to 0.66 (sd = 0.39) at 1 month (*p* < 0.01). Firmicutes had a prevalence of 100% with mean relative abundance of 0.45 (sd = 0.41). Its prevalence decreased from 0.65 (sd = 0.45) at 1 week to 0.32 (sd = 0.37) at 1 month (*p* < 0.01). The less dominant phyla were Bacteroidetes with prevalence 13% and relative abundance 0.02 (sd = 0.14) and Actinobacteria with prevalence 13% and relative abundance 0.002 (sd = 0.07). The most prevalent genera at 1 week were *Staphylococcus*, *Enterococcus* and *Klebsiella*, while *Escherichia*, *Shigella*, *Enterobacteriaceae* and *Enterococcus* are dominant at 1 month. The average Shannon diversity index increased over time from mean 0.38 (sd = 0.4) at 1 week, mean 0.71 (sd = 0.54) at 1 month, and mean 0.92 (sd = 0.44) at 2 months old (*p* < 0.01).

Patel, et al. [72] looked at 12 preterm gestation 28.2 ± 1.5 weeks, birthweight 1055 ± 234 g, inclusive of 1 NEC, 3 LONS. The rectal swab taken at birth and weekly showed a predominance of *Enterobacteriaceae* (Proteobacteria), *Staphylococcaceae* (Firmicutes) and *Enterococcaceae* (Firmicutes) with infrequent *Lactobacillaceae* and *Bifidobacterium* species. *Enterobacteriaceae* comprised 33% of bacterial families in week 1 and increased to 53% by week 3 to 5 (*p* = 0.021). *Enterobacteriaceae* accounted for 60% of Proteobacteria in week 1, 84% in week 2 and 88% by week 3 to 5. Proteobacteria comprised 55% at week 1 and 59% by week 3 to 5. At 1, 2, 3 weeks, Shannon was 1.4, 0.8, 0.9 and Chao1 was 15–18, 6–12, 7–11 respectively. The Shannon diversity was lowest at birth, increased by week 2 and declined rapidly by week 3 to 5 (*p* < 0.01).

In summary, the postnatal very preterm gut is colonised in a structured sequence by facultative anaerobes followed by obligate anaerobes. The initial class is Bacilli followed by a bloom of Gammaproteobacteria and finally an increase in Clostridia. There is a paucity of *Lactobacillus*, *Bifidobacillus* and the phyla Bacteroidetes and Actinobacteria. The final pattern is well established by 30 to 36 weeks corrected age. At phylum level, Firmicutes dominates at 1 week and Proteobacteria at 1 month. The prevalence of Firmicutes at 1 week is 0.65 decreasing to 0.32 by 1 month while Proteobacteria increases from 0.28 to 0.66 over the same period. The Shannon diversity index ranges from 0.38 to 1.4 at 1 week, increases by 2 weeks and reaches 0.71 to 0.9 by 1 month.

## 3. Factors Influencing Gut Microbiome of Preterm Neonate

The very preterm neonates are exposed to factors which alter the initial colonization and delay the maturation of the preterm gut microbiome. These factors include the bacteria causing chorioamnionitis and subsequently present in the meconium, the degree of prematurity, the delivery mode, delay and type of feeding, exposure to maternal and postnatal antibiotics and long stay in the neonatal wards.

### 3.1. Maternal Chorioamnionitis and Bacteria Derived from First Meconium

Puri, et al. [81] looked at the stools in the first 3 weeks in extremely preterm neonates with 32 chorioamnionitis, 26 chorioamnionitis with funisitis and 48 controls. Chorioamnionitis and chorioamnionitis-fusitis had higher LONS or death (*p* = 0.0033). Genus *Sneathia* and family *Mycoplasmaceae* were associated with higher risk of NEC, LONS or death (*p* = 0.01, OR 5.5, 95% CI 1.3, 21.0).

Chernikova, et al. [82] analysed the weekly stools of 9 extremely preterm with gestation 24–29 weeks, birthweight 510–1295 g, inclusive of 2 prolonged PPROM (pPPROM), 2 chorioamnionitis. pPPROM neonates had more *Staphylococcus* (*p* = 0.013) and *Streptococcus* (*p* = 0.033) and faster increase of *Enterobacter* (*p* = 0.009) and slower *Clostridium* (*p* = 0.02). Neonates with maternal chorioamnionitis showed faster increase of *Serratia* (*p* < 0.001) and *Parabacteroides* (*p* = 0.004).

For preterm neonates, there is one study on meconium [69] and numerous studies on meconium and stools [47,70,72,73,74,75,77,83]. None of these studies correlate the bacteria present in the meconium with the subsequent pattern of postnatal gut microbiome.

### 3.2. Gestational Age

La Rosa, et al. [73] stated that the rate of a structured maturation of gut microbiome was dependent on the gestation age at birth and postnatal age. The study by Chernikova, et al. [77] showed that the gestational age at birth had a significant influence on gut microbiome, and the Simpson index was 0.35 for extremely preterm versus 0.65 for very preterm neonates. Gregory, et al. [75] concurred that gestational age at birth drove the trajectory of microbiome development during the first 3 weeks of life. Drell, et al. [80] showed a positive correlation between Shannon index and corrected gestational age (*p* < 0.001).

### 3.3. Delivery Mode

The following studies on preterm neonates did not show any association between delivery mode and gut microbiome. Stewart, et al. [84] studied 25 spontaneous vaginal delivery (SVD) and 21 cesarean section (CS) preterm infants with gestation 24–31 weeks in the first 100 days of life. The birth mode was not associated with the relative abundance of genera, and both alpha (within) and beta (between) diversity. Grier, et al. [74] did a study on 95 preterm with gestation 28.8 ± 3.4 weeks and 25 term infants with 60 CS and 60 SVD. The delivery mode was not significantly associated with gut microbiome after controlling for age. In Patel, et al. [72] on 12 VLBW with gestation 28.2 ± 1.5 weeks, birth weight 1055 ± 234 g, 1 NEC, 3 LONS with 67% (8/12) CS, there was no association between mode of delivery and gut microbiome composition in VLBW infants managed in the NICU.

Several studies showed that the delivery mode in preterm neonates contributed to some differences in the gut microbiome. In Ho, et al. [79] on 45 VLBW infants with SVD 11/45 (24.4%), Gammaproteobacteria was positively associated with SVD in ≤2 weeks of life (F = 9.55, *p* = 0.002) while Firmicutes was positively associated with CS (F = 21.49, *p* < 0.001). A study by Wandro, et al. [76] on preterm VLBW neonates showed that *Bacteroides* was found in 4/9 SVD and none of the 22 CS. The mode of delivery accounted for 12% variance of the bacterial composition. Similarly, the study by Arboleya, et al. [83] on 27 gestation 24–32 weeks, 7 SVD, 20 CS showed that SVD babies had more *Bacteroides* at 10 days of life (*p* < 0.05).

### 3.4. Feeding—Type of Milk

Gregory, et al. [75] studied 3 groups of 10 preterm <32 weeks gestation per group, of predominant maternal breast milk (MBM), pasteurised donor human milk (PDHM) or infant formula (IF). The preterm gut microbiome was influenced by birthweight (*p* < 0.001), postnatal age (*p* < 0.001) and types of feeding (*p* < 0.001). The gut was dominated by Bacillales (Firmicutes) and Lactobacillales (Firmicutes) until 28 to 30 weeks corrected age especially in IF fed ELBW infants. MBM had more Clostridia (Firmicutes) and Enterobacteriales (Proteobacteria). The succession in MBM infants was Bacillales, Lactobacillales, then Enterobacteriales, Clostridiales and Bifidobacteriales. IF had higher persistent Bacillales and Lactobacillales. In IF, Clostridiales was 10 times more in VLBW compared to ELBW infants. The Shannon diversity of MBM increased from 2.2 to 2.8 and for IF from 2.1 to 2.9.

La Rosa, et al. [73] studied 58 preterm with 15 gestation <26 weeks, 20 gestation 26–28 weeks, 23 gestation >28 weeks. These neonates were fed on zero human milk 2 (3.4%), <10% human milk 16 (57.1%), 10–50% human milk 18 (31.0%), >50% human milk 22 (37.9%). Breast milk was associated with increased proportion of Gammaproteobacteria but not after 28 weeks gestation.

### 3.5. Antibiotics/Other Medications

Although intrapartum antibiotic prophylaxis aims to prevent EOS, particularly GBS infection, in neonates, perinatal antibiotics influence the types of bacteria present and diversity of the preterm gut microbiome. Arboleya, et al. [83] did a retrospective cohort study on 27 neonates with gestation 24–32 weeks with 13 full-term, vaginally delivered, exclusively breast-fed (FTVDBF) controls. The perinatal antibiotics including intrapartum antimicrobial prophylaxis resulted in an increase of *Enterobacteriaceae*. Meanwhile, significantly higher abundance of *Comamonadaceae* (Proteobacteria), *Staphylococcaceae* (Firmicutes) and Bacilli (Firmicutes) (*p* < 0.05) was observed in infants without exposed to antibiotics. In another study by Arboleya, et al. [47] demonstrated that antibiotic administration significantly reduced the *Bifidobacterium* levels in preterm neonates at 10 days of life (*p* < 0.05), but not on other microbial groups tested.

A similar result is obtained by Chernikova, et al. [77] on 17 NICU infants with antibiotic use 94%, 13 preterm, 176 term neonates. In premature infants, antibiotic use was associated with lower *Bifidobacterium* (*p* = 0.015) and *Bacteroides* (*p* = 0.041). The study by La Rosa, et al. [73] on 58 preterm neonates with median percent 14 (IQR 5.8,25.6) days of life on antibiotics, showed that antibiotic use was associated with increased proportion of Gammaproteobacteria for gestation ≥26 weeks and decreased Clostridia ≤28 weeks.

Dardas, et al. [85] analysed the rectal swab at 10 and 30 days in 29 neonates with gestation 24–32 weeks and duration of antibiotics 2 days versus 7 days. The neonatal gut microbiome was affected by postnatal age and antibiotic exposure. Firmicutes and Bacteroidetes predominated the 10-day sample. There was a rise in Proteobacteria and Actinobacteria at 30 days. For those neonates who received 7–10 days versus 2 days antibiotics, there was a significantly reduced Shannon index from 10 days of life after adjustment for delivery mode.

Wandro, et al. [76] studied 32 VLBW neonates with 25 on antibiotics and 6 not on antibiotic and one unknown status. The preterm gut microbiome was influenced by antibiotics irrespective of the health outcomes of being 21 healthy, 8 LONS and 3 NEC. Antibiotic use was associated with lower Shannon diversity (*p* = 0.06).

### 3.6. Environment

Ho, et al. [79] studied 45 VLBW infants with gestation 27.9 ± 2.2 weeks over one month. There were two distinct clusters based on the abundance of Gammaproteobacteria during the first 2 weeks. One group of 20 neonates had decreasing Firmicutes and low Gammaproteobacterial which slowly increased with time. A second group of 25 neonates had high Gammaproteobacterial and low Firmicutes throughout. A single variant of *Klebsiella* dominated in the second group suggesting an environmental origin of the gut microbiome. The study suggested the dominance of *Klebsiella* could be due to the repeated exposure to the hospital environment among the women with high-risk pregnancies who had received inpatient care before giving birth [79].

Brooks, et al. [78] followed only 2 preterm gestion 26 and 28 weeks with faeces collected every 3 days for 1 month. One neonate had facultative anaerobes for 12 days before shift to obligate anaerobes. The second neonate had facultative anaerobes throughout. The dominant gut taxa are also present in room environment namely *Staphylococcus epidermidis*, *K. pneumoniae*, *Bacteroides fragilis* and *E. coli*. This suggested the environment was a source of the microbes in the premature gut. Brooks, et al. [86] did a metagenomic study on the microbes present in 50 preterm neonates and the environment of the NICU. The common strains present in the environment and gut were *Staphylococcal epidermidis*, *Enterococcus faecalis*, *Pseudomonas aeruginosa* and *Klebsiella pneumoniae*. These microbes were present in the environment before and after detection in the preterm gut.

Taft, et al. [87] studied preterm neonates <29 weeks with 33 LONS and 33 controls in 2 sites. The gut microbiome disruption before the LONS was depending on the postnatal age and site. This implied that the environmental microbiome had an influence on the gut microbiome.

## 4. Gut Bacteria in NEC

NEC is a life-threatening intestinal disease with mortality as high as 30%, affecting 5–10% of preterm infants [35]. It involves severe inflammation and necrosis of the intestines with features characteristic of pneumatosis intestinalis [88]. The risk and mortality of NEC increases with lower gestation age [89]. Despite extensive ongoing research, the exact role of the gut microbiome in the pathophysiology of NEC is still elusive. NEC may occur in the presence of gut dysbacteriosis with low bacterial diversity and one pathogen group dominates the disease process.

### 4.1. The Pathogens Associated with NEC

There are a few studies which seem to implicate a pathogen or group of pathogens causes NEC, but this finding is not replicated in other studies. de la Cochetiere, et al. [90] performed 16S rRNA studies on preterm <30 weeks gestation with 3 NEC and 9 controls. The stools were collected at 1st day and weekly. *Clostridium perfringens* was detected in the first 2 weeks of life in 3 neonates who later developed NEC. The controls did not have *Clostridium perfringens*. On the other hand, Heida, et al. [91] investigated the bacterial invasion of 43 surgical NEC specimens compared to 43 age-matched controls. Using FISH probes technique, *Enterobacteriaceae* dominated the NEC specimens.

### 4.2. Abundance of Proteobacteria and Low Abundance of Firmicutes Is Associated with NEC

Several studies seem to indicate that NEC occurs when there is an abundance of Proteobacteria with concomitant reduction of Firmicutes. Warner, et al. [92] performed a nested case control within a cohort VLBW study of 46 NEC with 120 controls. There was an increased proportion of facultative anaerobic Gammaproteobacteia (Proteobacteria) (*p* = 0.0011) and decreased proportions of strict anaerobic Negativicutes (Firmicutes) (*p* = 0.0013) and Clostridia-Negativicutes (both Firmicutes) (*p*= 0.005) in NEC compared to controls. The Gammaproteobacteria comprised mainly *Enterobacteriaceae*, *Escherichia*, *Shigella* and *Klebsiella*. The Negativicutes were mainly *Veillonella*, *Negaticoccus* and *Megasphaera*.

Claud, et al. [26] studied the gut microbiome development in preterm 5 NEC (mean gestation 26.8 weeks) and 5 matched controls (mean gestation 26.4 weeks). Weekly stool samples were collected for 10 weeks. Three weeks before NEC, the cases had an increase of Proteobacteria and decrease of Firmicutes (especially *Veillonella*, *Peptococcaceae*). The controls did not have such disruption.

Mai, et al. [93] did a matched case-control nested in a cohort study with 9 NEC and 9 controls, gestation ≤32 weeks, and birth weight ≤1250 g. NEC had lower Actinobacteria and Bacteroidetes compared to controls. In the week before NEC, Proteobacteria increased from 36.2 to 70.9% (34% increase) and Firmicutes decreased from 60.7 to 28.8% (32% decrease). For the controls, there was no significant change in phyla over time.

In a study by Wang, et al. [94] on 10 NEC gestation 25–31 weeks versus 10 controls gestation 26–32 weeks, the stool sample at NEC diagnosis (average collection time <1 day after diagnosis) was analysed. The controls had 4 phyla, namely Firmicutes (relative abundance 57.8%), Proteobacteria (35.0%), Bacteroidetes (2.5%) and Fusobacteria (0.5%), unclassified bacteria (4.3%). The NEC cases had only 2 phyla, namely, Proteobacteria (90.8%), Firmicutes (9.1%), unclassified bacteria (0.16%). The NEC cases compared to controls had an increase in Proteobacteria and decrease in Firmicutes (*p* < 0.001).

### 4.3. Abundance of Both Proteobacteria and Firmicutes Is Associated with NEC

Torrazza, et al. [95] did a study on preterm neonates ≤32 weeks with 18 NEC and 35 controls. The first meconium and then weekly stool samples were analysed. Two weeks before NEC, cases compared to controls had higher Proteobacteria 61% versus 19%. One week before NEC, cases compared to controls had higher Actinobacteria 3% versus 0.4% respectively. NEC neonates had lower *Bifidobacteria* and Bacteroidetes in the weeks preceding diagnosis. The proportion of Firmicutes (*Clostridia*, *Lactobacilli*) increased from 34% from 2 weeks to 52% from 1 week, to 72% just before NEC compared to less increase of Firmicutes in controls. This increase in Firmicutes in cases was not seen in other studies described previously. There was no difference between cases and controls for within sample α-diversity Chao1 at 2 weeks, 1 week and 0 week before diagnosis.

Sim, et al. [96] studied 12 NEC with 44 controls in preterm neonates <32 weeks. Seven out of 11 NEC had abundance of *Klebsiella* (Proteobacteria) (*p* = 0.049) and 4/11 NEC had abundance of *Clostridia* (Firmicutes) (*p* = 0.006).

### 4.4. Firmicutes Disruption Is Associated with NEC

McMurtry, et al. [97] recruited 95 preterm neonates ≤1500 g, gestation ≤34 weeks with 21 NEC and 74 controls. Unlike other NEC publication, the researcher classified the 21 NEC cases into 8 mild, 7 severe, 6 lethal grades of NEC. The total NEC cases versus controls showed less of the following bacteria, namely Actinobacteria (*p* = 0.009), lower *Clostridia* (*p* = 0.004), *Veillonella* (*p* = 0.007) and *Streptococcus* (*p* = 0.002). There was no difference in Bacilli and Gammaproteobacteria. The total NEC compared to controls has lower Chao1 (*p* < 0.0001) and Shannon’s (*p* = 0.0002). Comparing the grades of NEC to controls, the average abundance of *Clostridia* was 12% in mild NEC, 3% in severe NEC and 0% in lethal NEC. The Clostridia abundance in lethal NEC was lower than mild NEC (*p* = 0.025). As for the 74 controls, 14 (19%) lacked *Clostridia* while 12/21 (57%) of total NEC lacked *Clostridia* (*p* = 0.002). This study seemed to imply Clostridia (Firmicutes) was protective of NEC.

Stewart, et al. [98] studied 38 infants recruited by convenient sampling with median 27 weeks gestation and median birthweight 895 g. Eight developed NEC (4 surgical) and 13 had LONS. First and weekly stools were obtained and analysed using standard culture method and 16S technique. Standard culture showed the most common bacteria were *Enterococcus faecalis* 40% and coagulase negative staphylococci (CONS) 39%. NEC versus healthy infants had more CONS (Firmicutes) (45% versus 30%) and less *Enterococcus faecalis* (Firmicutes) (31% versus 57%). Using 16S technique, *Enterococcus* was associated with NEC and *Staphylococcus* with LONS.

### 4.5. Association of Gut Dysbacteriosis with NEC

The published studies do not support the concept that NEC consistently arises from the worst gut dysbacteriosis. Morrow, et al. [99] recruited preterm neonates <29 weeks gestation including 11 NEC with 21 matched controls. There were two distinct forms of dysbacteriosis prior to NEC. The NEC were either preceded by Firmicutes relative abundance ≥98% from day 4 to 9 or Proteobacteria (specifically *Enterobacteriaceae*) relative abundance ≥90% from days 10 to 16 days. The predictive value was 88% (*p* < 0.001) as only 25% of controls have this phenotype. All NEC cases lacked *Propionibacterium* (Actinobacterium).

Stewart, et al. [100] enrolled 318 preterm <32 weeks gestation of which had 7 NEC (median gestation 26 weeks (range 23–30 weeks)) matched with 28 controls (median gestation 27 weeks (range 24–30 weeks)). The dominant bacteria in all samples were *Klebsiella*, *Escherichia*, *Staphylococcus* and *Enterococcus*. There were 6 distinct clusters of preterm gut community types each dominated by *Klebsiella*, *Klebsiella and Enterococcus*, *Staphylococcus*, *Enterococcus*, *Escherichia*, mixed population with more *Bifidobacterium*. There was no causative pathogen detected in NEC, but more *Bifidobacterium* may protect against NEC.

Normann, et al. [101] studied 10 NEC with 16 matched controls in extremely low birthweight neonates. There was no difference in the weekly stool microbiome and bacterial diversity in patients developing NEC and controls. The predominant bacteria were *Enterococcus* (Firmicutes), Bacillales (Firmicutes) and *Enterobacteriaceae* (Proteobacteria). Prior to NEC, the cases had more Bacillales and *Enterobacteriaceae* and the controls had more *Enterococcus* (all were not statistically significant). In the 16 controls, the *Enterococcus* and Bacillales were succeeded by *Enterobacteriaceae*.

Mshvildadze, et al. [71] looked at 23 preterm neonates with gestation 23–32 weeks, birthweight 520–1997 g, inclusive of 6 NEC. The overall profiles in NEC were not distinguishable from controls. NEC showed an increase of *Citrobacter*-like (Proteobacteria) and *Enterococcus*-like (Firmicutes) sequences compared to controls. Controls had more frequent *Klebsiella* (Proteobacteria) (*p* = 0.06) and *Enterobacteriaceae* (Proteobacteria) (*p* < 0.05) which differed from other studies.

### 4.6. Bacterial Diversity of NEC Versus Non-NEC Patients

NEC seems to occur in preterm gut microbiome with lower bacterial diversity. Studies which show lower bacterial diversity are Wang, et al. [94], McMurtry, et al. [97] and Morrow, et al. [99]. In McMurtry, et al. [97], the median Shannon diversity in the 74 controls was 3.5 compared to 2.5 in the 21 NEC (*p* < 0.001). The difference was significant with mild (*p* = 0.01), severe (*p* = 0.03) and lethal (*p* = 0.04) NEC, compared to controls. The relative lack of diversity in the infants developing NEC has been attributed to the fact that they were receiving more days of antibiotic therapy prior to manifestation of NEC when compared to normal infants [94].

Nevertheless, there were studies which do not show any difference, such as Mai, et al. [93], Torrazza, et al. [95] and Normann, et al. [101]. Pammi, et al. [35] did a meta-analysis on 8 studies with 106 NEC and 278 controls. The alpha Shannon 2.8 and beta Simpson 0.75 diversity indices were similar for NEC and controls.

In summary, majority of the study demonstrated that microbial dysbacteriosis preceding NEC in preterm infants. Furthermore, the association between the increase in abundance from the phylum Proteobacteria while decrease in Firmicutes and Bacteroidetes with the predisposition to NEC in preterm infants was well supported by a systematic review and meta-analysis based on sequence data collected from previous studies [35]. Even though that intestinal dysbacteriosis being the major factor in NEC pathogenesis, the specific role of the microorganisms and their interactions with host immune system is yet to be clearly elucidated. That said, the fact that higher relative abundance of Proteobacteria presents as a cause or consequence of NEC is still elusive. Considering that Proteobacteria are gram negative microbes possessing large quantities of lipopolysaccharide (LPS) on their cell walls, an immune interaction mediated by toll-like receptor (TLR) 4 induction has been proposed as a potential mechanism inducing NEC. In fact, over-expression of TLR4 is observed in premature infant gut that involved in maintaining the equilibrium between adequate inflammatory response and homeostasis [102]. Thus, together with underdeveloped adaptive immunity of preterm infants and an increase in Proteobacteria may trigger excessive pro-inflammatory response in preterm gut, causing intestinal injury and necrosis [103].

## 5. Gut Bacteria in Late-Onset Neonatal Sepsis (LONS)

Late-onset neonatal sepsis remains a serious complication of prematurity that occurs between day 7 and day 10 after birth [104]. In general, empiric antibiotics are given to pregnant women or preterm infants to reduce the risk of EOS. Paradoxically, gut microbiome is disrupted as a result of the antibiotic therapy, giving way to the expansion and dominance of opportunistic pathobionts in the premature infants, hence leading to LONS [105].

A few studies showed a change in gut microbiome pattern before LONS. Mai, et al. [30] did a nested case control study in preterm ≤32 weeks with 10 LONS cases and 18 controls. The first meconium and weekly stools were analysed. The author postulated that there was a lack of normal gut microbiome rather than the presence of potential pathogens as a risk factor for LONS and NEC [72]. There was a delay in colonisation by Proteobacteria in the LONS cases. Two weeks before LONS, the cases had lower Proteobacteria 0.5% compared to 20% in controls. Just before LONS, the cases experienced a late bloom of Proteobacteria to 15% compared to a decrease to 9.8% in controls. There was a higher proportion of Firmicutes in LONS two weeks before the diagnosis. There was less *Bifidobacteria* in cases compared to controls.

Carl, et al. [28] studied 163 VLBW neonates which included 11 LONS cases. The bacteria causing septicaemia were GBS × 4, *Serratia marcescens* × 2, *Escherichia coli* × 2, *Klebsiella pneumoniae* × 1, *MRSA* × 2. Seven out of 11 (64%) neonates had GBS × 3, *S. marcescens* × 2, *E. coli* × 2 in their stool before their septicaemia. The common origin of the matched faecal and blood isolates was confirmed by genomic sequencing. The *E. coli* appeared earlier in the stool while the GBS and *S. marcescens* were detected closer to the septicaemia.

Shaw, et al. [29] studied preterm neonates with gestation <32 weeks with 22 LONS and 44 matched controls and daily faecal samples were collected. The most abundant OTUs (90%) in faeces closest to the time of LONS were *Klebsiella*, *Escherichia*, *Staphylococcus*, *Enterococcus* and *Clostridium*. Enterobacteriaceal and staphylococcal LONS were associated with the bacterial OTUs in their stools.

Taft, et al. [87] studied preterm neonates <29 weeks in 2 sites. There were 13 and 20 LONS cases plus same number of controls in the 2 sites (NICU). The gut microbiome disruption occurred before the LONS, but the distortion depended on postnatal age and the site. Bacterial sequencing could detect the sepsis-causative organism in the stool in 82% of LONS.

To sum up, several gram-positive and gram-negative enteric bacteria have been identified as the major pathobionts causing LONS in preterm infants, including *Klebsiella* spp., *E. coli* and *Serratia* spp. (Gram-negative) and the *Streptococcus* spp., *Enterococcus* spp. and CONS. Furthermore, the LONS has also been associated with perturbed preterm gut microbiome, with lower bacterial diversity, higher abundance of Proteobacteria and Firmicutes but low in *Bifidobacteria*. Interestingly, these causative microorganisms were shown to be intestinal origin rather than microbial translocation from the skin as demonstrated by stool sampling from LONS cases.

## 6. Conclusions

Gut microbiome has been reported to be associated with various human diseases [106,107,108,109,110,111]. The pattern and magnitude of the bacterial gut disruption is an active ongoing research subject in preterm infants with adverse outcomes. However, there is still a wide knowledge gap on how the presence of gut bacteria at birth either alone or in combination with early postnatal gut dysbacteriosis interact to contribute to the mortality and severe pre-discharge morbidities of very preterm infants. In particular, the mechanistic information on how specific microbiota patterns might lead to NEC or LONS in preterm infants is still lacking. Thus, an improved understanding of the diversity of microbes causing NEC and LONS may propel forward important research regarding their sources, pathogenic mechanisms, microbiome-host interactions and clinical implications. Perhaps, different strategies of altering the neonatal microbiome to promote favorable microbial profile may prevent NEC and LONS in the high-risk preterm neonates. Furthermore, continued research into the empiric antibiotic usage in NICU is recommended in order to have a better-informed antibiotic selection in clinical practice. Therefore, these potential insights will be important for developing improved prevention, diagnostic and treatment strategies for NEC and LONS.
